# Access to Health Care Services for Afghan Refugees in Iran in the COVID-19 Pandemic

**DOI:** 10.1017/dmp.2020.240

**Published:** 2020-07-14

**Authors:** Ibrahim Salmani, Hamed Seddighi, Maryam Nikfard

**Affiliations:** Department of Health in Disaster and Emergency, School of Public Health, Shahid Sadoughi University of Medical Sciences, Yazd, Iran; Student Research Committee, University of Social Welfare and Rehabilitation Sciences, Tehran, Iran

**Keywords:** COVID-19, health equality, refugees

Islamic Republic of Iran is among the top 10 countries most affected by coronavirus disease (COVID-19) worldwide.^1^ The first case of the disease was diagnosed on February 19, 2020, in Iran and spread rapidly throughout the country.^1^ The latest statistics of COVID-19 in Iran on June 20 indicate that the total number of people living with COVID-19 in Iran reached 202 584, the death toll reached 9507, and the total number of people recovered from the disease so far reached 161 384.^2^ The Iranian Government took various measures to respond, including closing schools, universities, and guilds. In addition, the Iranian Government decided to shut down most sectors during the Iranian New Year holidays in March 2020 for 2 weeks.^3^ The refugees are a critical population regarding diseases control.^4^ According to the United Nations High Commissioner for Refugees (UNHCR), Iran is one of the 10 countries hosting the largest number of refugees in the world.^5^ Afghan nationals form the largest population of refugees in Iran.^6^ Sharing a common culture, religion, and language with Iranians has raised the number of refugees from Afghanistan in the last 40 years.^6^ According to the statistics, more than 2 million Afghan nationals live in Iran, of whom 40% are registered with asylum cards, 2% hold long-term residence permits, 22% possess short-term passports, and 36% live in Iran with no official documents.^7^ In Iran, given the problems that Afghan refugees face, special attention needs to be made to this population in the COVID-19 response. The first and ever-existing challenge is the lack of insurance and, consequently, high medical expenses. Many cannot afford insurance due to lack of residence cards, which results in not referring to medical centers and the unwillingness to be hospitalized. Considering the fact that most refugees in Iran are day laborers and do not have an income due to the COVID-19 outbreak and nationwide quarantine, the number of patients referred to clinics has decreased. Therefore, the main concern for treatment is related to the medical expenses that, if solved, there will be no problem in hospitalizing these people. More than 124 000 (6%) Afghan nationals in Iran have registered for health insurance.^7^ The refugees can be insured in 2 ways: the social security insurance that gives the same services to the refugees as what is provided for the Iranians, and a specific health insurance for the refugees with asylum cards. Afghan nationals work in a variety of service occupations ranging from high-paying self-employers to low-income laborers with an average income of US$ 150 per month. Considering the fact that a noticeable number of wage workers have lost their jobs as a result of the COVID-19 pandemic and the challenge of crowded families, economic problems have been exacerbated for the refugees.

In Iran, the basic service package is provided free-of-charge through Universal Health Coverage plan for 95% of the population.^8^ In general, health insurance covers 70% of the costs of insurer-listed drugs, as well as 90% of public hospital costs, with extra provision for those with rare diseases or in remote areas.^9^ Typical costs of COVID-19 for patients range from US$ 30 to 1600, depending on the patient’s condition and need for critical care. Hospital services and testing are provided free of charge for COVID-19 patients. Other challenges can be mentioned as low level of education and consequently low health literacy, lower access to cyberspace, and not following COVID-19 news which might be responsible for observing less safety and health procedures by the refugees. Moreover, as a result of low health literacy and lack of awareness of diseases, refugees have always hesitated to refer to the medical centers even in cases of serious illnesses. Iranian health houses provide free and quality primary health care services for the refugees in the settlements as for the whole population in all cities of Iran, which solves the lack of access to health services.^10^ However, the problem lies in the cultural belief that stops the refugees to refer to the health centers and care about the health issues being particularly observed in the illiterate, the middle-aged and above, and undocumented refugees. Despite poor sanitation facilities in the houses, the younger generation has taken a different approach and is more concerned about the health issues. A large number of refugees in Iran are second or third generations living in the country.^11^

As the next drawback, a majority of refugees may live in groups in crowded living conditions in different parts of the city, especially in old areas and abandoned houses, which makes it difficult to control these people in the outbreaks, hence the need for accurate planning for monitoring. According to UNHCR, approximately 97% of the refugees live in urban and semi-urban areas, whereas the remaining 3% reside in refugee settlements^12^ – although the exact percentage is yet to be known for undocumented refugees.

Given the above, it seems necessary for the Iranian Government to take appropriate actions to assist the refugees. In this regard, a facilitating point in Iran is sharing the same language with Afghan nationals. Full coverage of all refugees (legal and illegal) with health insurance seems to be essential. Advocacy in international assemblies to attract financial resources can be considered as a way to finance this issue. Free-of-charge treatment for infected refugees can be an alternative to reassuring refugees until full insurance coverage is provided. Despite the high costs for the government, health coverage should save on total health expenditures over the long run, facilitating the control over the patients and avoiding the rapid spread of disease to a larger population. Other necessary measures to maintain refugees’ health are listed as follows: raising refugees’ awareness through community-based programs, involving refugees’ representatives in relevant decisions, distributing essential items for prevention (eg, masks and disinfectants), supporting them with their livelihood to reduce traffic, and avoiding forced repatriation. In conclusion, improved access to affordable care for the refugees might be considered as a way to make all of Iran safer from COVID-19.
